# Supplementary material for: Lipidomic Analysis of Cells and Extracellular Vesicles from High- and Low-Metastatic Triple-Negative Breast Cancer
Source: Metabolites. 2020 Feb 13;10(2):67. doi: 10.3390/metabo10020067 (PMC7073695; doi:10.3390/metabo10020067)
Supplement: Supplementary file 1 [file metabolites-10-00067-s001.zip › Supplementary materials/Supplementary materials_Figures S1-S4.pdf]

Supplemental Materials

Lipidomic analysis of cells and extracellular vesicles from high- and low-metastatic triple-negative breast cancer

Nao Nishida-Aoki[1,†], Yoshihiro Izumi[2,*], Hiroaki Takeda[2,‡], Masatomo Takahashi[2], Takahiro Ochiya[1,3,*] and Takeshi Bamba[2]

[1] Division of Molecular and Cellular Medicine, National Cancer Center Research Institute, Chuo-ku, Tokyo 104-0045, Japan; naoki@fredhutch.org (N.N.-A)

[2] Division of Metabolomics, Medical Institute of Bioregulation, Kyushu University, Higashi-ku, Fukuoka 812-8582, Japan; hiroaki.takeda@riken.jp (H.T.); m-takahashi@bioreg.kyushu-u.ac.jp (M.T.); bamba@bioreg.kyushu-u.ac.jp (T.B.)

[3] Department of Molecular and Cellular Medicine, Institute of Medical Science, Tokyo Medical University, Shinjuku-ku, Tokyo 160-8402, Japan

* Correspondence: izumi@bioreg.kyushu-u.ac.jp (Y.I.); tochiya@tokyo-med.ac.jp (T.O.)

[†] Present address: Division of Human Biology, Fred Hutchinson Cancer Research Center, Seattle, WA, 98109-1024, USA

[‡] Present address: Laboratory for Neural Cell Dynamics, RIKEN Center for Brain Science 2-1 Hirosawa, Wako-shi, Saitama 351-0198, Japan

A

D3H1	Total particles (x10^{11})	Total protein (μg)
1	3.01	112
2	3.01	118
3	1.02	103.8

D3H2LN	Total particles (x10^{11})	Total protein (μg)
1	1.38	94.8
2	2.7	120
3	2.4	95.4

B

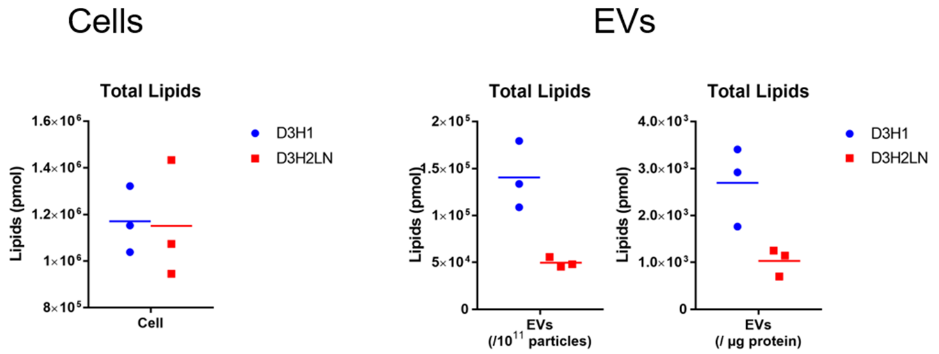

C

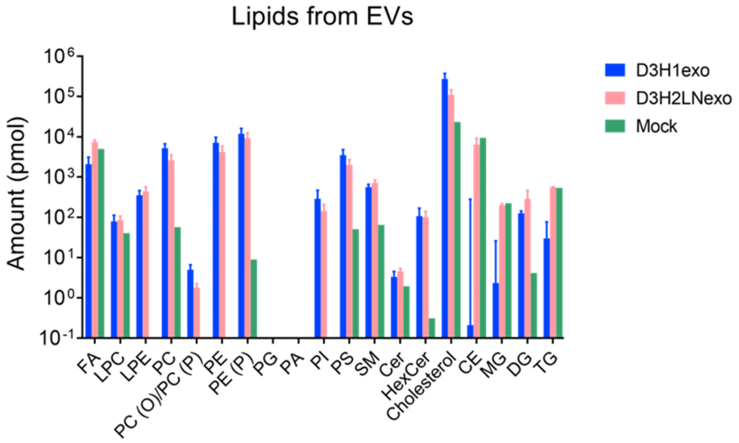

Figure S1. Details of cellular and EV samples from D3H1 and D3H2LN. (A) The amount of isolated EVs based on particle numbers and protein amounts from D3H1 and D3H2LN in 3 independent replicates. (B) The sum of all lipid species detected in cellular and EV samples. For EVs, total lipids were normalized by particle numbers (middle) or protein amount (right). The bars indicate average of the 3 datapoints. (C) Absolute amount of lipids detected from D3H1 EVs, D3H2LN EVs before subtraction of background and normalization, and mock samples. Some lipid species were detected in the mock sample which was collected with the medium incubated without cells and processed correspondingly as EV collection. The mol amount of the detected individual lipid species in the mock sample was subtracted from the mol of EVs as background to calculate the actual lipid mol in EVs.

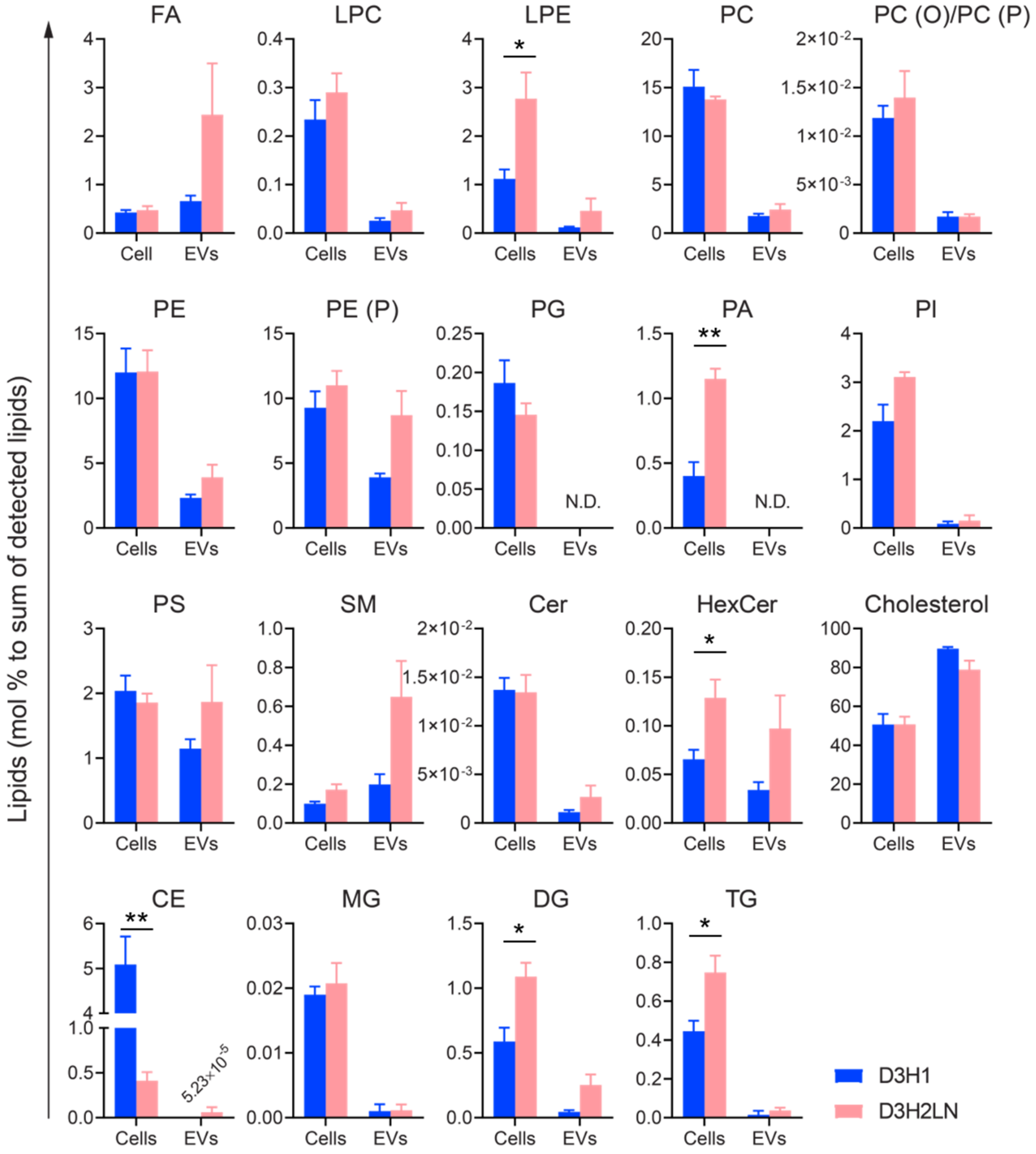

Figure S2. Comparison of mol percentile of lipid classes between D3H1 and D3H2LN in cells and EVs. The mol percentile of each lipid classes (the same data with Figure 2A) was depicted in bar graphs. The error bars depict ± SEM of 3 independent replicates. N.D. indicates not detected. *** p<0.001, ** p<0.01, * p<0.05, Student’s t-test (unpaired, two-tailed)

A

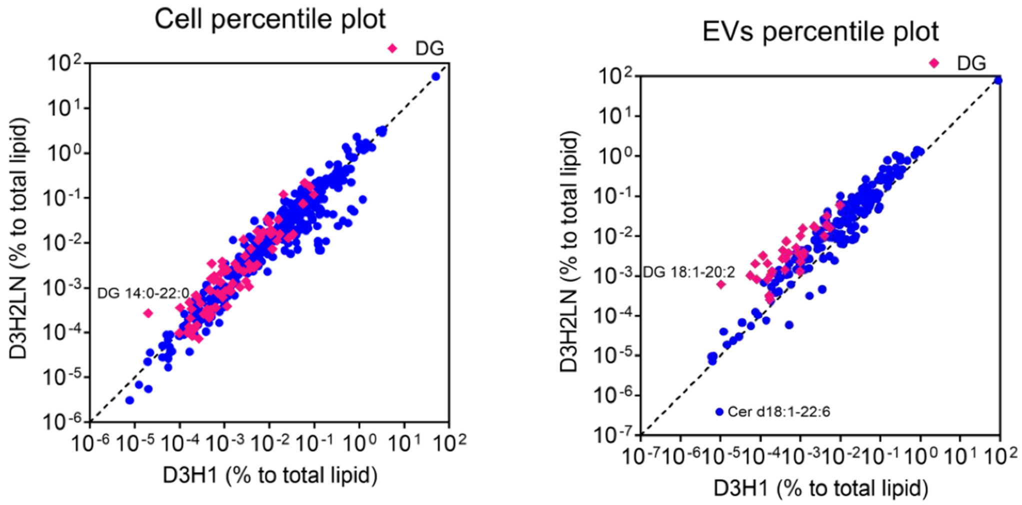

B

D3H1 EVs-specific	D3H2LN EVs-specific
LPC 18:2 (sn-2)	SM d18:1-12:0
LPC 20:0	HexCer d18:1-14:0
LPC 18:2 (sn-1)	CE 20:5
Cer d18:1-22:6	TG 46:0
LPC 18:1 (sn-1)	TG 48:2
	TG 50:1
	TG 50:2
	TG 52:2
	TG 52:5

Figure S3. Comparison of individual lipid species. (A) Scatter plots of mol percentile of lipids in D3H1 and D3H2LN. Left: Cells, Right: EVs. The hatched lines indicate the percentage of D3H1 and D3H2LN is the same. (B) List of lipids only detected in EVs from either D3H1 or D3H3LN. (C) The mol quantities of individual lipid species in cells and EVs of D3H1 and D3H2LN. Error bars indicate mean ± SEM. N.D. indicates not detected. *** $p<0.001$, ** $p<0.01$, * $p<0.05$, Student's t-test (unpaired, two-tailed)

Figure S3. (Continued)

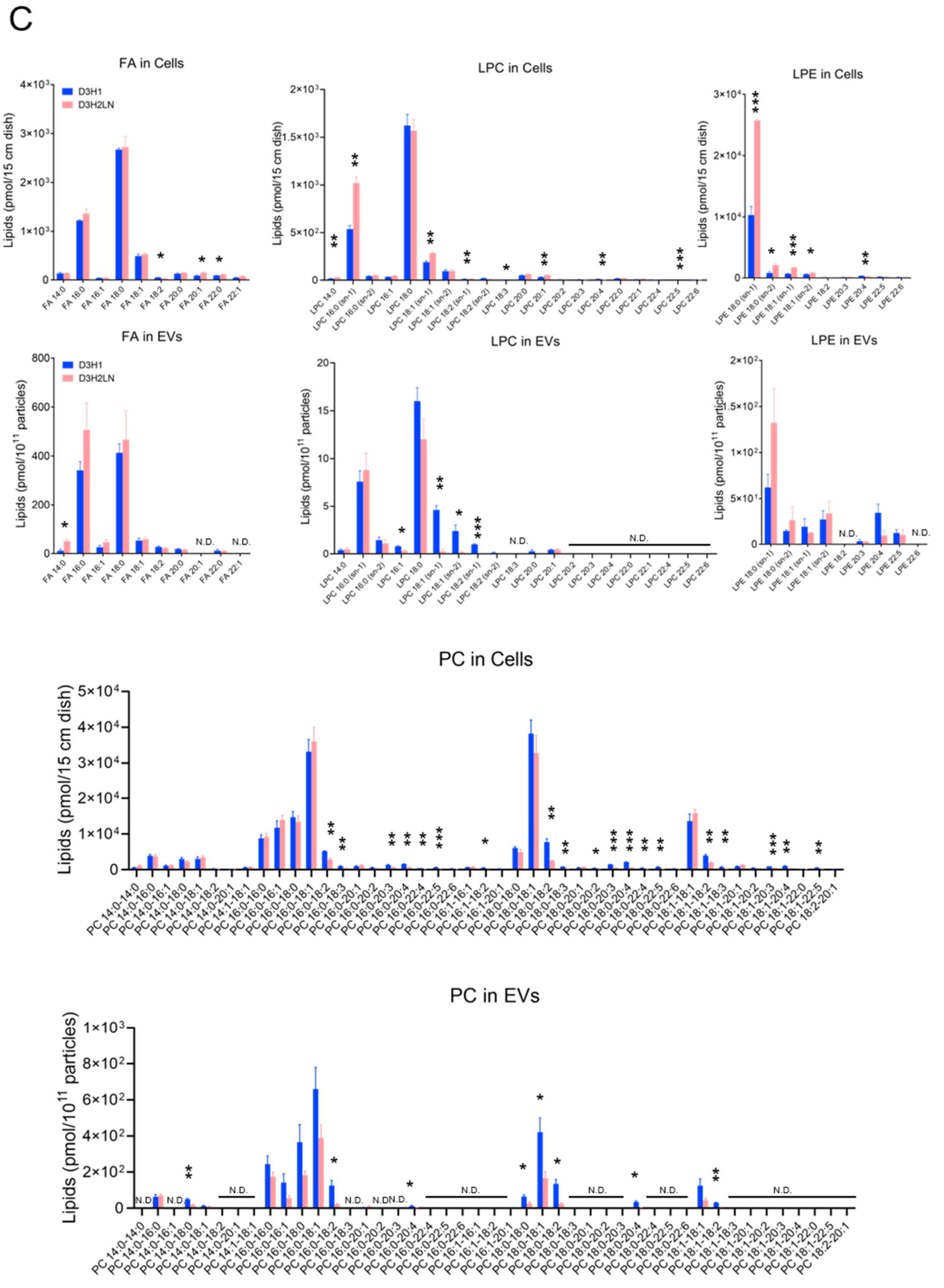

Figure S3. (Continued)

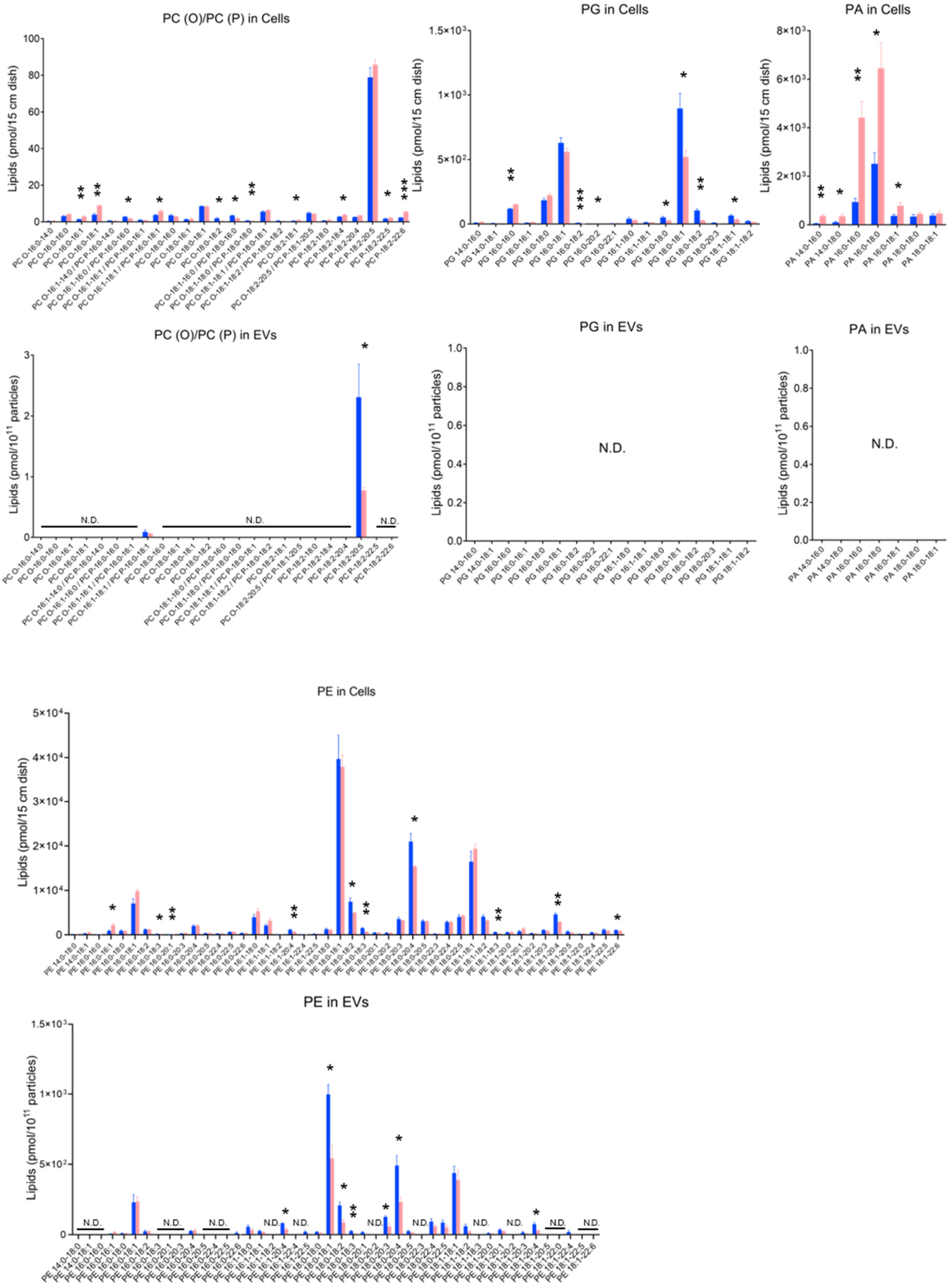

Figure S3. (Continued)

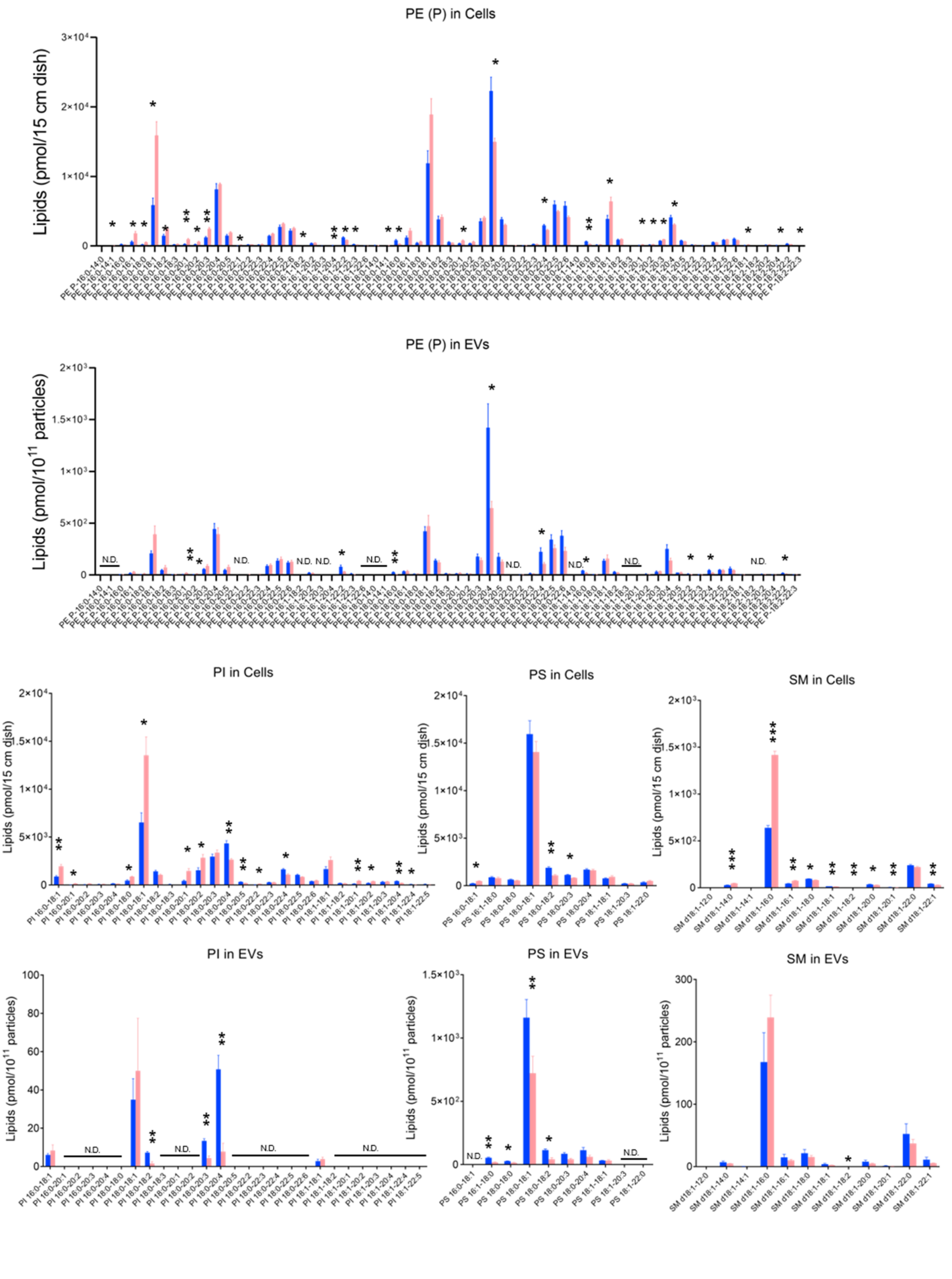

Figure S3. (Continued)

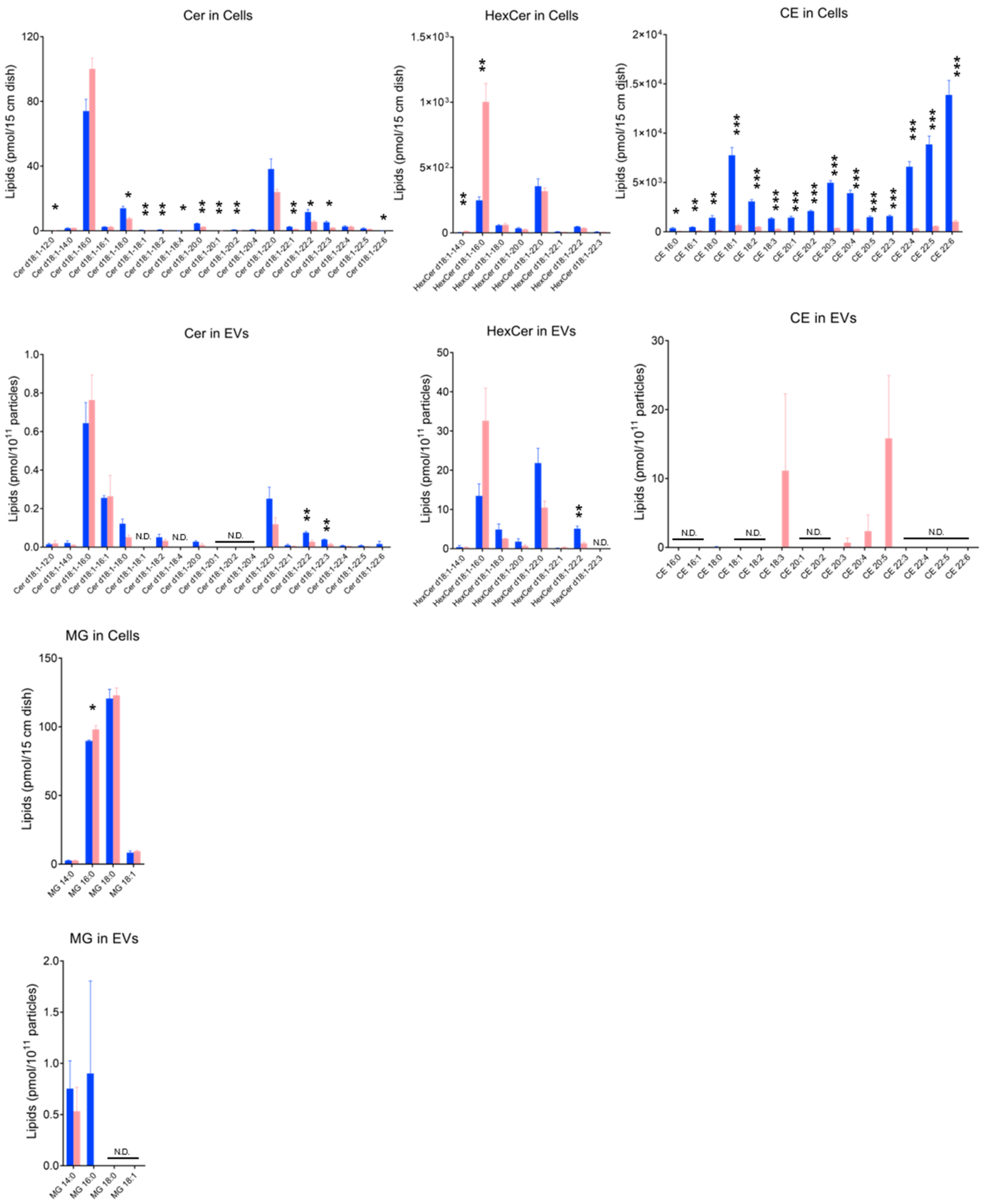

Figure S3. (Continued)

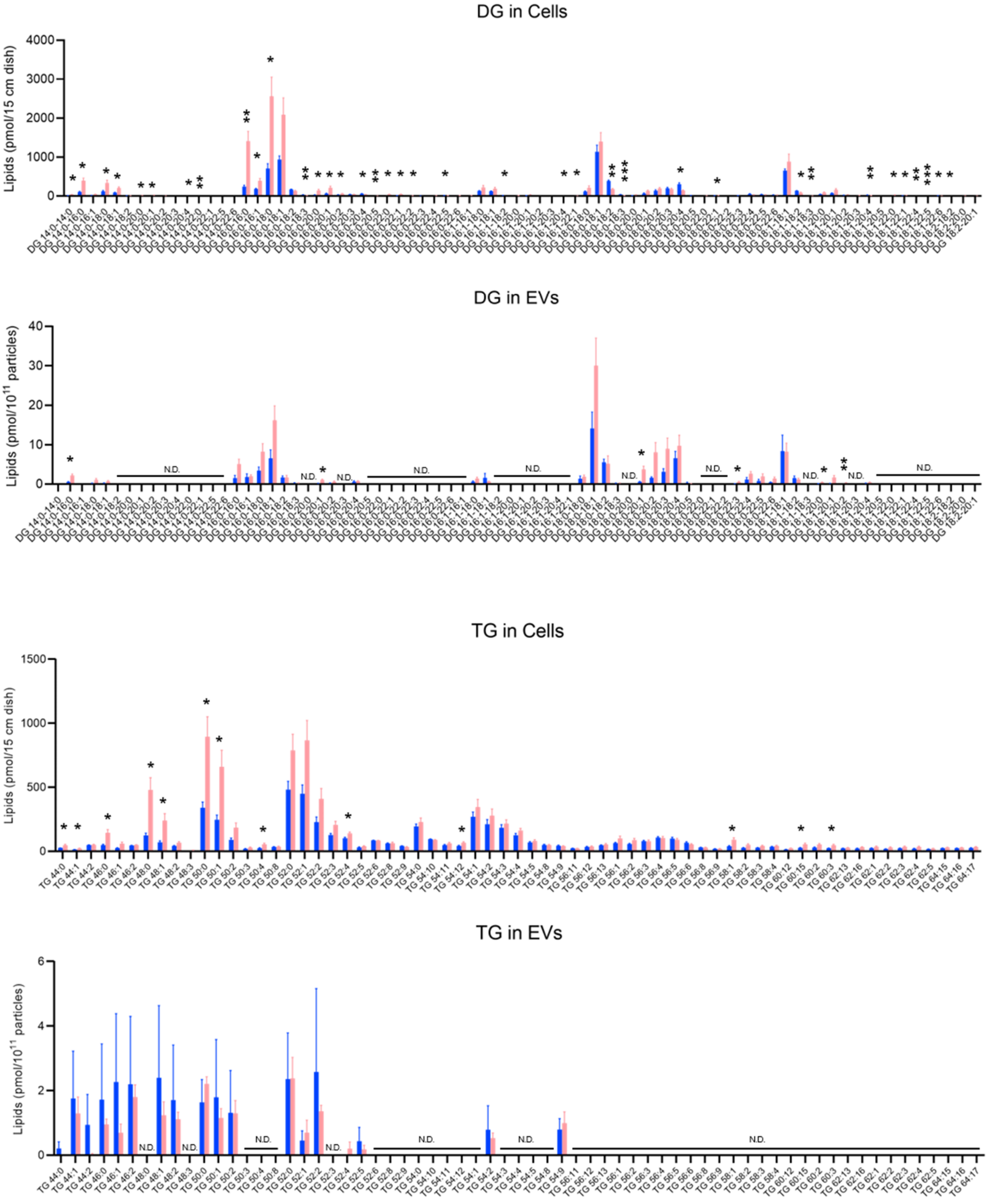

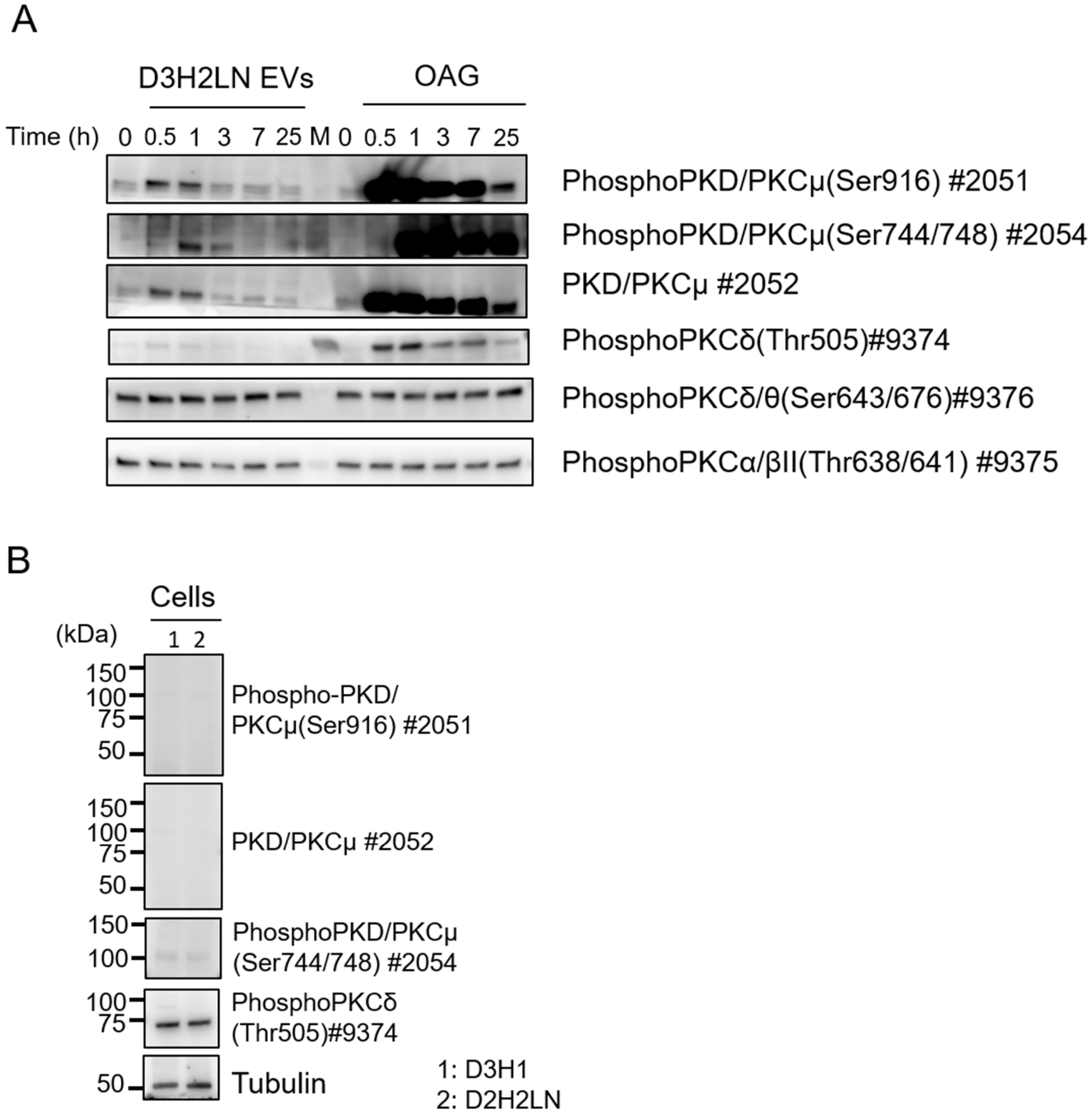

Figure S4. Phosphorylation levels of PKC proteins in HUVECs and TNBC cell lines. (A) Immunoblot of time-dependent phosphorylation levels of PKC proteins in HUVECs treated by EVs from D3H2LN or OAG. HUVECs were incubated with EVs or OAG for the indicated time. Proteins were detected using the indicated antibodies. The lane labeled with M depicts the loading marker. (B) Phosphorylation levels of PKC in D3H1 and D3H2LN cell lysates. Proteins were detected using the indicated antibodies. Blots were detected using a high-sensitive detection dye.
